# miR-524-5p suppresses the growth of oncogenic BRAF melanoma by targeting BRAF and ERK2

**DOI:** 10.18632/oncotarget.2452

**Published:** 2014-09-08

**Authors:** Szu-Mam Liu, Jean Lu, Hoong-Chien Lee, Feng-Hsiang Chung, Nianhan Ma

**Affiliations:** ^1^ Institute of Systems Biology and Bioinformatics, National Central University, Jhongli, Taiwan; ^2^ Genomics Research Center, Academia Sinica, Taipei, Taiwan; ^3^ Center for Dynamical Biomarkers and Translational Medicine, National Central University, Jhongli, Taiwan; ^4^ Department of Physics, Chung Yuan Christian University, Jhongli, Taiwan

**Keywords:** miR-524-5p, melanoma, BRAF, ERK2, MAPK signaling, microRNA

## Abstract

It has been well documented that miRNAs can modulate the effectiveness of cancer-associated signaling pathways. Mitogen-activated protein kinase (MAPK/ERK) signaling plays an essential role in the progression of many cancers, including melanoma and colon cancers. However, no single miRNA is reported to directly target multiple components of the MAPK/ERK pathway. We performed a miRNA PCR array screening with various MAPK/ERK signaling activities. The miRNA array data revealed that the expression of miR-524-5p was decreased in cells with an active MAPK/ERK pathway and confirmed that the expression of miR-524-5p is inversely associated with the activity of the MAPK/ERK pathway. We demonstrated that miR-524-5p directly binds to the 3′-untranslated regions of both BRAFandERK2 and suppresses the expression of these proteins. Because BRAF and ERK2 are the main components of MAPK signaling, the overexpression of miR-524-5p effectively inhibits MAPK/ERK signaling, tumor proliferation, and melanoma cell migration. Moreover, tumors overexpressing miR-524-5p were significantly smaller than those of the negative control mice. Our findings provide new insight into the role of miR-524-5p as an important miRNA that negatively regulates the MAPK/ERK signaling pathway, suggesting that miR-524-5p could be a potent therapeutic candidate for melanoma treatment.

## INTRODUCTION

miRNAs are small non-coding RNA sequences that are approximately 19-25 nucleotides in length. The discovery of these molecules has revolutionized our understanding of the post-transcriptional regulation of gene expression. Mature miRNAs bind to one or more mRNAs and interfere with gene expression by either affecting mRNA stability or by interfering with protein translation [1, 2]. miRNAs modulate gene expression via base-pairing between the seed sequence of the miRNA (nucleotides 2-8 at its 5′ end) and its complementary seed match sequence in the 3′-untranslatated region (UTR) of the target mRNA [3]. Functional studies have predicted that miRNAs can control the activity of over 30% of all protein-coding genes [4]. Thus far, more than 2000 mature miRNAs have been identified in humans, regulating numerous biological functions, including development, tumor formation, and immune responses. Moreover, miRNAs may act as oncomirs or as tumor suppressors, may be used in diagnostics or as prognostic biomarkers, and have been regarded as very promising therapeutic targets [5-7].

miRNAs have been identified as a new class of modifiers of signal transduction pathways [8-10]. They could directly target signaling pathways to affect the outcome of the pathway or to coordinate among signaling pathways [11]. Current research has shed light on the mechanisms by which miRNAs regulate signaling pathways via a single miRNA or multiple miRNAs. A single miRNA may suppress one or more mRNAs in a common signal transduction pathway, and several miRNAs may cooperatively or redundantly regulate a single signaling pathway. More interestingly, miRNAs may be involved in the feedback loops of gene regulation networks and confer robustness to signaling pathways [12]. For instance, miR-301 can be induced by NF-κB signaling in pancreatic cancer cells to target NF-κB repressing factor (NKRF), which in turn inhibits NF-κB signaling in pancreatic cancer [13]. Nonetheless, how miRNAs regulate signaling pathway networks remains largely unknown.

The MAPK/ERK signaling pathway is a highly conserved intercellular signaling system present in multicellular organisms and plays an essential role in cancer progression. MAPK/ERK activation is a common feature of tumors with KRAS, NRAS, or BRAF mutations [14, 15]. For instance, a highly activated MAPK/ERK pathway is found in approximately 30% of cancers and over 60% of melanomas, and it is associated with tumor proliferation and migration [16-19]. BRAF is upstream of the MAPK/ERK pathway, and a single amino acid change, resulting in a valine to glutamic acid substitution at position 600 (V600E), accounts for ~90% of BRAF mutations. This dominant activating mutation of V600E *BRAF* is responsible for 50-70% of melanomas. Numerous reports have suggested that the inhibition of V600E *BRAF* signaling blocks melanoma cell proliferation and induces apoptosis *in vitro* and *in vivo* [20, 21]. Previous studies have shown that protein phosphorylation and dephosphorylation and the association of proteins with scaffolds and adaptors provide temporal and spatial regulation of the MAPK/ERK pathway. However, many regulatory mechanisms of the MAPK/ERK pathway remain undefined [22, 23]. Indeed, there is little information linking miRNAs to the MAPK/ERK signaling pathway, and there is no published evidence for miRNAs directly targeting BRAF, MEK, or ERK, the main components of the MAPK/ERK pathway in melanoma [6, 24].

In this study, we performed a miRNA screen and verified that the expression of miR-524-5p is down-regulated in cells with an activated (BRAF mutated) MAPK/ERK pathway but not in wild-type BRAF cells. We further show that BRAF and ERK2 are the targets of miR-524-5p. This is the first finding demonstrating that BRAF can be regulated by miRNA and that a single miRNA can target MAPK/ERK signals at two different components. The overexpression of miR-524-5p suppresses the cell proliferation, migration, and transformation induced by an activated MAPK/ERK pathway in melanoma cells by down-regulating the MAPK/ERK pathway. Our novel findings show that miR-524-5p expression is regulated via the MAPK/ERK pathway and that miR-524-5p functions in a feedback mechanism to inhibit MAPK/ERK signaling through BRAF and ERK2 in melanoma progression.

## RESULTS

### Expression of miR-214, miR-433, and miR-524-5p is reduced in melanoma cells

**Figure 1:**
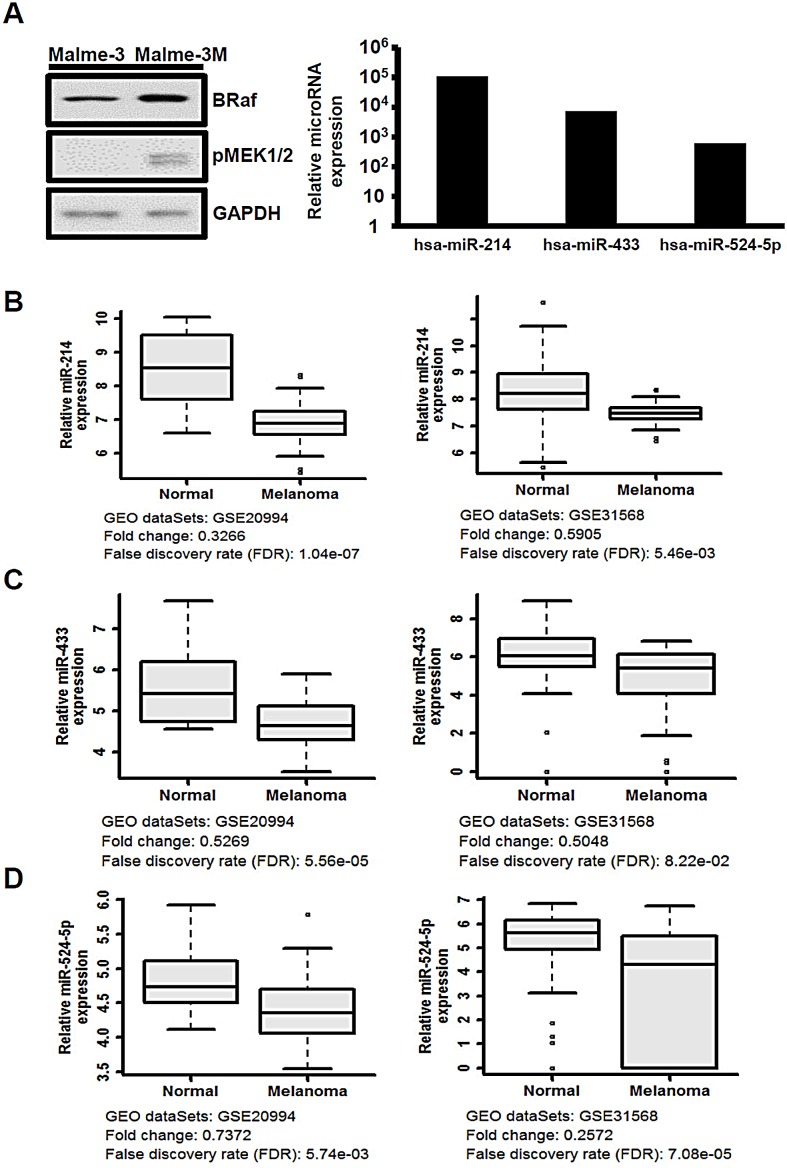
The expression of miR-214, miR-433, and miR-524-5p is suppressed in melanoma (A) Left panel, MAPK/ERK signaling was highly activated in the Malme-3M cell line according to Western analysis to detect the protein levels of BRAF and phospho-MEK. Right panel, the relative expression levels of miR-214, miR-433, and miR-524-5p were detected by the TaqMan miRNA expression array (normalized to RNU44 and RNU48) with the ratio of Malme-3 to Malme-3M. (B-D) Box-whisker plots of miR-214, miR-433, and miR-524-5p in melanoma samples. The miRNA expression profiles were obtained from the Gene Expression Omnibus (GEO) accession numbers GSE20994 and GSE31568. We downloaded raw data and used statistical analysis to obtain the mean and FDR values. The fold change was evaluated from the mean of melanoma samples versus normal.

Malme-3 and Malme-3M cells were collected from the same patient and represent normal epithelial-bearing wild-type BRAF and melanoma cells with the V600E *BRAF* mutation, respectively. These two cell lines are often used as the cell model in comparative *in vitro* melanoma studies and provide tumor and normal counterparts for activation of MAPK/ERK pathway signaling (Figure 1A). We performed a screen using a high-throughput quantitative real-time miRNA PCR array to investigate the difference in the expression levels of a total of 366 miRNAs between Malme-3 and Malme-3M. The expression of 216 miRNAs could be detected in both cell lines, and we compared their expression profiles (Supplementary Table 1). Interestingly, we found that the miRNA levels are globally repressed in tumor cells, which is consistent with previous observations [25]. The expression levels of 30 miRNAs were up-regulated over 500-fold, and the expression levels of 3 miRNAs were down-regulated over 100-fold in the Malme-3 versus Malme-3M cells. Among them, the observed miR let-7a levels confirm the prediction that the expression of miR let-7a is reduced in activated MAPK/ERK cells, as it is known that miR let-7a directly targets NRAS of the MAPK/ERK signaling pathway (Supplementary Table 1).

We further assessed the significant correlations of the 33 miRNAs in melanoma patients. To compare the expression of these miRNAs in normal skin and melanoma tissues, we analyzed the public patient microarray data from the GEO database. Two genome-wide miRNA expression profiles from 22 normal controls and 35 melanoma cancer samples (GEO accn.: GSE20994) and 70 normal controls and 35 melanoma cancer samples (GEO accn.: GSE31568) were analyzed by microarrays [26, 27]. We noted that only three miRNAs, miR-214, miR-433, and miR-524-5p, exhibited relative expression similar to that observed in our screen of Malme-3 versus Malme-3M cells (Figure 1A, right panel). Despite of the different materials and methods used, the expression levels in primary melanoma were consistently much lower than those in normal specimens (Figure 1B-D). Previous studies have reported that both miR-214 and miR-433 are associated with the MAPK/ERK pathway; miR-214 is the regulator of ERK1/2, and miR-433 is the regulator of Runx2 [28-30]. However, there are no reports implicating miR-524-5p in the MAPK/ERK pathway. Thus, we further investigated the possible role of miR-524-5p in MAPK/ERK signaling in melanoma.

### Expression of miR-524-5p is associated with the MAPK/ERK pathway

**Figure 2:**
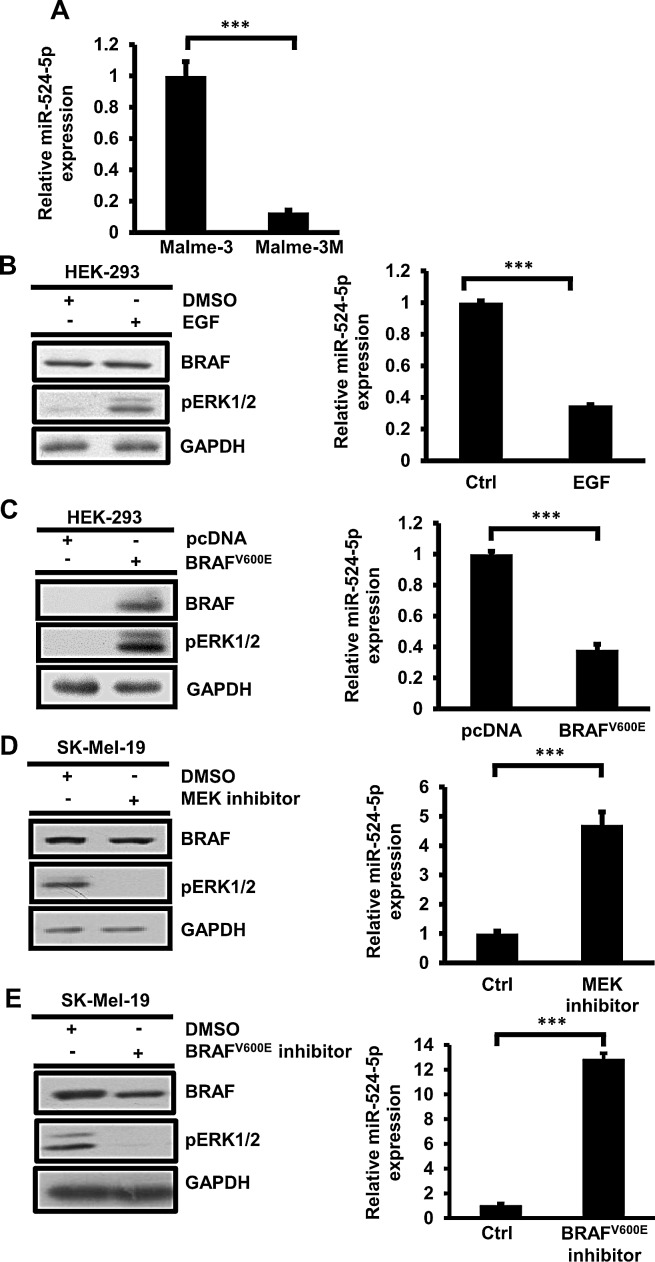
miR-524-5p expression is down-regulated in activated MAPK/ERK signaling (A) Endogenous expression of miR-524-5p in Malme-3 and Malme-3M cells was determined by quantitative RT-PCR. (B)-(E) Left panel, the activity of the MAPK/ERK pathway was measured by Western blotting to detect the protein levels of BRAF and phospho-ERK. Right panel, the expression level of miR-524-5p was detected by quantitative RT-PCR (normalized to RNU48). (B) Activation of MAPK signaling by treatment with EGF for 2 hours in HEK293 cells. (C) Over-expressing mutated V600E BRAF in HEK293 cells for 48 hours resulted in activation of the MAPK pathway and the down-regulation of miR-524-5p expression. (D) SK-Mel-19 cells were treated with or without 50 nM of MEK1/2 inhibitors PD0325901 for 24 hours, and the cells were collected for analyses. (E) SK-Mel-19 cells were treated with or without 5 μM of V600E BRAF inhibitors PLX4032 for 8 hours, and the cells were collected for analyses. (A-E) Each value represents the average from triplicates. Error bars mark the standard deviations (Student's *t*- test: ^***^ *p*< 0.001)

We verified the endogenous expression of miR-524-5p in Malme-3 and Malme-3M cells by quantitative real-time PCR. Indeed, the expression of miR-524-5p in Malme-3 cells was 8.3-fold higher than in Malme-3M cells (Figure 2A). We investigated whether the low expression of miR-524-5p is associated with the activation of MAPK/ERK signaling. To do so, we treated HEK293 cells with EGF or by overexpressing mutated V600E *BRAF* to activate MAPK/ERK signaling. The expression of miR-524-5p was significantly reduced when MAPK/ERK was activated (Figure 2B and 2C). Interestingly, after MAPK/ERK activation either by EGF treatment or overexpression of mutated V600E *BRAF*, the expression of miR-524-5p in HEK293 cells decreased by approximately 2.6-fold. A similar reduction of miR-524-5p was observed by overexpressing mutated V600E *BRAF* in SK-Mel-187 cells with endogenous wild-type BRAF (Supplementary Figure 1A) [19]. We further addressed whether the expression level of miR-524-5p is affected by inhibiting hyperactivation of the MAPK/ERK pathway. We used either the specific MEK1/2 inhibitor PD325901 or V600E BRAF inhibitor PLX4032 to block highly activated BRAF signaling in SK-Mel-19 or A375 cells. We then examined the expression level of miR-524-5p. As shown in Figure 2D and 2E, and Supplementary Figure 1B and 1C, the expression level of miR-524-5p was significantly increased when MAPK/ERK signaling was blocked. The same increase in the expression of miR-524-5p was observed by applying another specific MEK1/2 inhibitor, U0126, thus ruling out the non-specific effect of the inhibitors (data not shown). This result implies that the expression level of miR-524-5p is associated with activity of the MAPK/ERK pathway.

### miR-524-5p directly inhibits the expression of BRAF and ERK2 through their 3′UTRs

**Figure 3:**
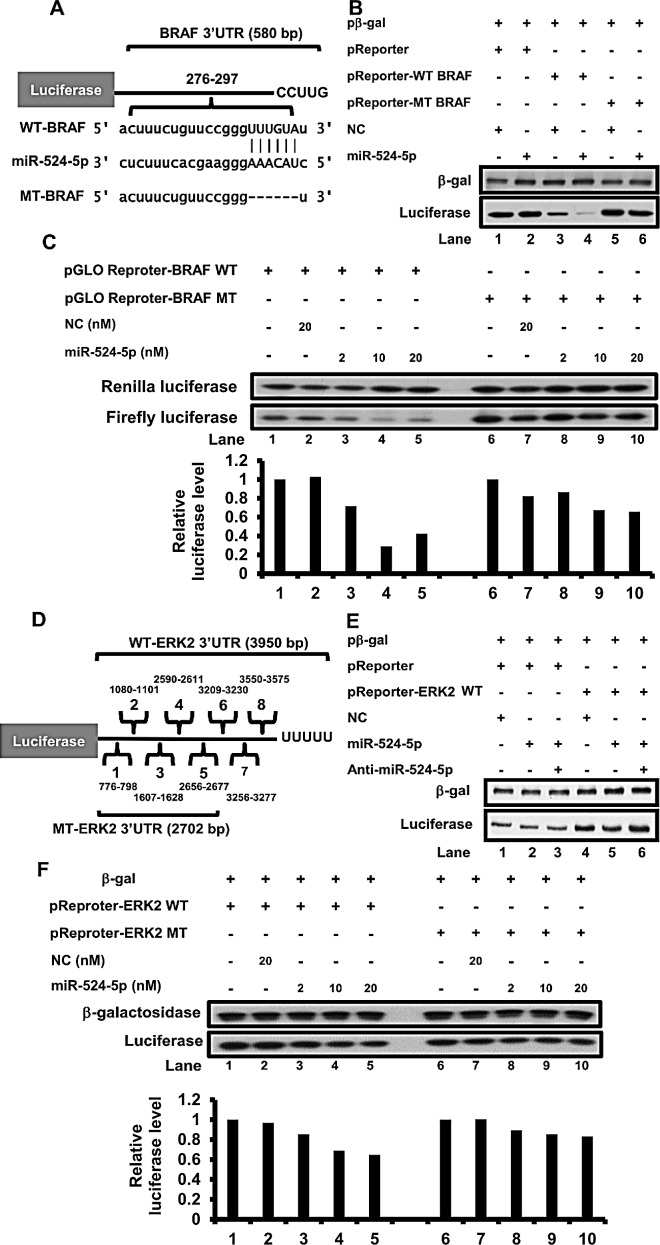
miR-524-5p represses BRAF and ERK2 expression (A) Schematic diagram of the BRAF reporter construct. BRAF harbors a miR-524-5p binding site within 276-297 nucleotides of the BRAF 3′-UTR. The wild-type BRAF 3′UTR contains a native miR-524-5p binding site; a mutant 3′UTR contains mutations that delete the seed match sequence of miR-524-5p. (B) Transfection of either the wild-type or mutant reporter into HEK293 cells led to the expression of luciferase. The expression level of luciferase was determined by immunoblotting after transfection for 48 hours. The transfection efficiency was normalized to the expression of -galactosidase. (C) Transfection of either 2, 10 or 20 nM mimic miR-524-5p with either the wild-type or mutant pGLO BRAF reporter into HEK293 cells led to the expression of Firefly and Renilla luciferase. The expression level was determined by immunoblotting after transfection for 48 hours. The transfection efficiency was normalized to the expression of Renilla luciferase. The lower panel is the relative qualification value of expression levels of Firefly luciferase versus Renilla luciferase. (D) Schematic diagram of the ERK2 3′-UTR reporter construct. Eight of miR-524-5p binding sites are predicted in the 3′ UTR of the ERK2 mRNA; a mutant 3′UTR of ERK2 reporter contains mutations that delete the 3′end sequence of three potential binding sites of miR-524-5p. (E) HEK293 cells were co-transfected with a luciferase reporter plasmid containing the wild-type ERK2 3′UTR and with miR-524-5p or both miR-524-5p and anti-miR-524-5p. (F) Transfection of either 2, 10 or 20 nM mimic miR-524-5p with either the wild-type or mutant ERK2 reporter into HEK293 cells led to the expression of luciferase. The transfection efficiency was normalized to the expression of β-galactosidase. The lower panel is the relative qualification value of expression levels of luciferase versus β-galactosidase.

To explore the relationship between miR-524-5p and the MAPK/ERK signaling pathway, we sought to identify the specific gene targets of miR-524-5p. Through bioinformatics analyses, we determined that the 3′-UTRs of human BRAF and ERK2 contain a match of the seed sequence of miR-524-5p (microRNA.org); one and eight potential miR-524-5p binding sites were located in the 3′ UTR of BRAF and ERK2, respectively (Figure 3A and 3D, and Supplementary Figure 2A). We constructed luciferase reporter vectors containing a fragment of the wild type 3′-UTR of BRAF or ERK2 to test whether BRAF or ERK2 is a direct target of miR-524-5p. The overexpression of miR-524-5p led to a decrease in the luciferase expression levels (Figure 3B and 3E). Most importantly, the suppression effect was alleviated when the miR-524-5p binding site was deleted from the 3′-UTR of BRAF (Figure 3B, Lanes 3 to 6). Increasing the concentration of miR-524-5p resulted in a consistent dosage-dependent attenuation of the expression of a luciferase reporter containing the wild type 3′-UTR of BRAF but not the mutant 3′-UTR of BRAF (Figure 3C). This indicated that binding of miR-524-5p to the seed region of BRAF is important for blocking BRAF expression.

We also further examined the suppression of ERK2 expression by miR-524-5p. As shown in Figure 3E, anti-miR-524-5p (miR-524-5p inhibitor) reversed the inhibition of luciferase expression in the ERK2 reporter (Figure 3E, Lanes 4 to 6). To confirm whether potential miR-524-5p binding sites in the 3′ UTR of ERK2 were important, we deleted three potential miR-524-5p binding sites at the 3′end of the 3′UTR of ERK2 to construct the mutant ERK2 3′UTR luciferase reporter vector. The result showed that the suppression of the luciferase expression levels were enhanced upon expression of miR-524-5p, but this effect was alleviated in cells with the mutant ERK2 3′UTR luciferase reporter (Figure 3F). Although increasing the concentration of miR-524-5p led to a slight decrease in luciferase levels in the cells expressing the mutant ERK2 3′UTR reporter, this might be due to effects from the other potential miR-524-5p binding sites. In summary, our results suggested that the 3′end of the 3′UTR of ERK2 contains miR-524-5p binding sites that are important for blocking ERK2 expression.

**Figure 4:**
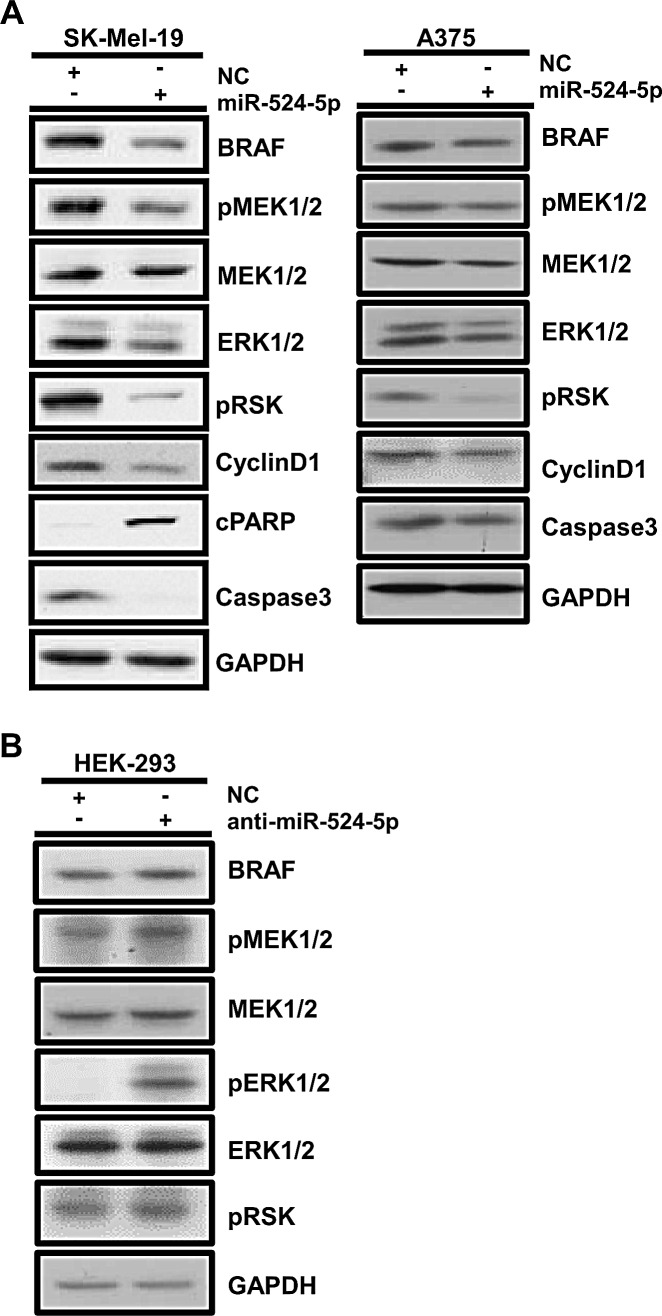
miR-524-5p regulates the MAPK/ERK signaling pathway (A) SK-Mel-19 or A375 cells were transfected with negative control (NC) or mimic miR-524-5p (10 nM applied in SK-Mel-19; 50 nM applied in A375), and cell lysates were collected at 48 hours for immunoblotting. (B) HEK293 cells were transfected with negative control (NC) or anti-miR-524-5p (20 nM), and cell lysates were collected at 48 hours for immunoblotting. GAPDH served as an internal control.

It is noted that when we transfected miR-524-5p into SK-Mel-19, A375 or Malme-3M cells, the expression levels of endogenous BRAF and ERK2 were reduced (Figure 4A and Supplementary Figure 2B). Taken together, these results indicate that miR-524-5p exerts inhibitory effects on BRAF and ERK2 expression via interaction with their respective 3′-UTRs.

### miR-524-5p reduces the activity of the MAPK/ERK pathway and regulates cell cycle and apoptosis proteins

As documented above, miR-524-5p can directly target the BRAF and ERK2 3′UTRs and can inhibit the translation of their cognate transcripts. Because BRAF and ERK are the main components of MAPK/ERK signaling, we wished to examine whether the overexpression of miR-524-5p can suppress MAPK/ERK signaling and its downstream events. As shown in Figure 4A, miR-524-5p overexpression in SK-Mel-19 or A375 cells, which exhibit highly activated MAPK/ERK signaling, suppressed the phosphorylation status of MEK and RSK. In addition, the overexpression of anti-miR-524-5p in HEK 293 cells, which have low endogenous MAPK/ERK activity, enhanced the phosphorylation status of MEK, ERK, and RSK and resulted in increased MAPK/ERK signaling (Figure 4B). This demonstrated that miR-524-5p can regulate MAPK/ERK activity. It has been documented that activated MEK/ERK signaling in melanoma with mutant BRAF cells enhances cell proliferation and inhibits apoptosis [31]. Indeed, levels of the cell proliferation marker cyclin D1 were reduced when miR-524-5p was overexpressed in SK-Mel-19 or A375 cells (Figure 4A). Furthermore, levels of the apoptotic maker cleaved poly (ADP-ribose) polymerase (cPARP) were increased and those of inactive caspase 3 were decreased. These results indicated that the overexpression of miR-524-5p attenuates MAPK/ERK signaling and its downstream targets, such as cyclin D1 and inactive caspase 3.

### miR-524-5p suppresses cell proliferation, growth, and migration

**Figure 5:**
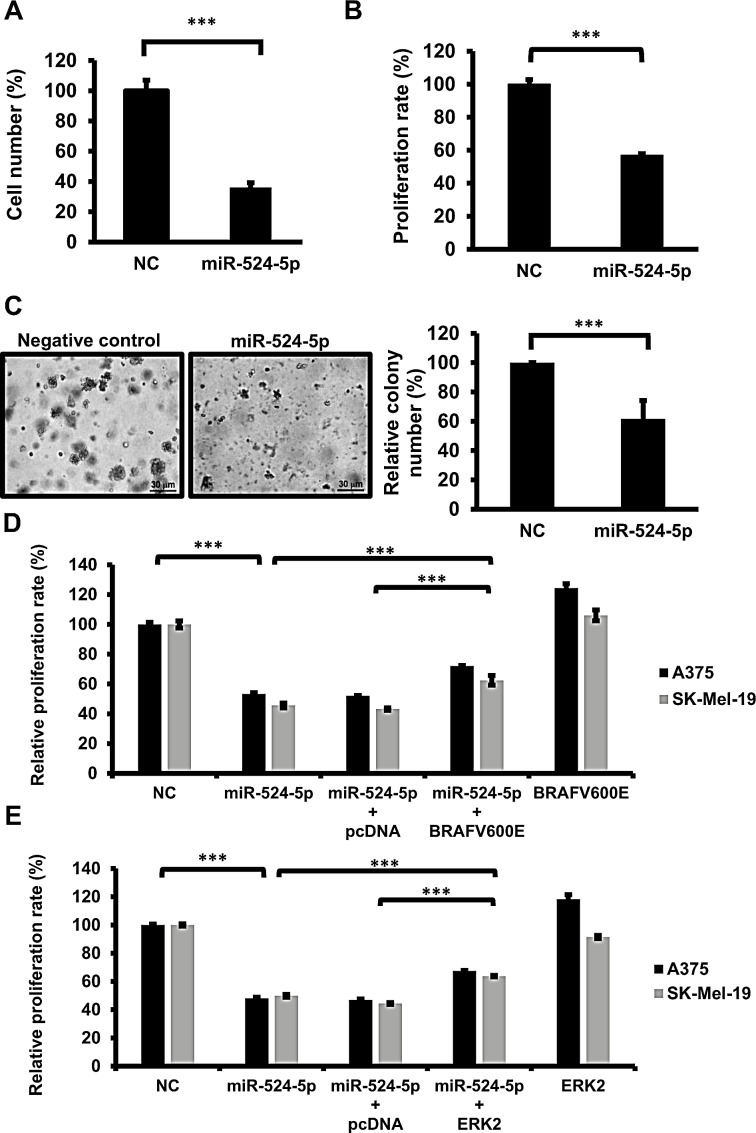
miR-524-5p suppresses cell proliferation and anchorage-independent growth in melanoma SK-Mel-19 cells were transfected with 10 nM negative control (NC) or mimic miR-524-5p. After 48 hours, the total cell number was calculated using a hemocytometer (A), and the proliferation activities were detected by the AlamarBlue assay (B). (C) SK-Mel-19 cells were transfected with a miR-524-5p expression plasmid or control vector. The ability to form colonies in soft agar represents the capacity for anchorage-independent growth. After 3 weeks, the number of colonies was calculated (Right panel), and the size of colonies was observed under microscopy (×50 magnification; Left panel). (D) and (E) The proliferation activities in SK-Mel-19 or A375, following transfection of cells with miR-524-5p or/with BRAF (D) or ERK2 (E) were measured. (A-E) All of the quantification data are reported as the mean ± SD of three independent experiments. (Student's *t*- test: ^***^ *p*< 0.001).

MAPK/ERK signaling plays a key role in mediating melanoma development, including the induction of cell growth and proliferation. Our results showed that miR-524-5p suppresses MAPK/ERK signaling through the down-regulation of BRAF and ERK. To gain insight into the functional roles of miR-524-5p in highly activated MAPK/ERK tumor cells, we investigated whether miR-524-5p affects cell growth and proliferation in melanoma cells *in vitro*. We transfected miR-524-5p into SK-Mel-19 cells for 2 days and detected their survival and proliferation rates. miR-524-5p could reduce the cell number more than 50% compared with the control miRNA (Figure 5A). AlamarBlue assays showed that the proliferation rate decreased by 40% in cells overexpressing miR-524-5p (Figure 5B). Moreover, consistent with the effects on cell number and proliferation, miR-524-5p significantly decreased the anchorage-independent growth of melanoma cells in soft agar compared with the negative control (Figure 5C). These results demonstrated that miR-524-5p expression can suppress the rates of cell survival, proliferation, and tumorigenesis activity *in vitro*. To confirm whether the growth inhibitory effect of miR-524-5p is due to its effect on BRAF and ERK2 expression, we co-expressed miR-524-5p with plasmids expressing BRAF or ERK2, without their corresponding 3′-UTRs. We observed that overexpression of BRAFV600E or ERK2 significantly rescued the inhibition of cell proliferation by miR-524-5p by approximately 20% in either SK-Mel-19 or A375 cells (Figure 5D and 5E). This further establishes a functional connection between miR-524-5p and BRAF or ERK2.

**Figure 6:**
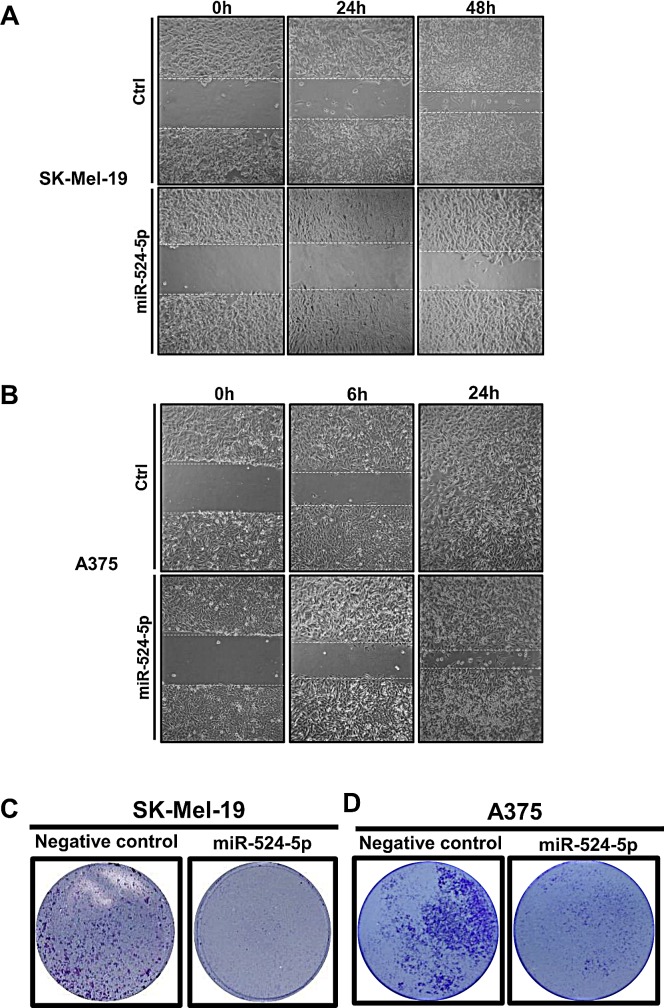
miR-524-5p reduces the migration of melanoma cells (A) and (B) Representative micrographs show the migration activity of cells after scratching in SK-Mel-19 (A) or A375 (B). At different time points, the wound healing activity was observed. (C) and (D) Cells were plated onto transwell filters, and the migration activity was detected by transwell assays. Representative images of stained filters are shown in SK-Mel-19 after 48 hours (C) or A375 after 24 hours (D).

Moreover, it has been suggested that the V600E *BRAF* mutation enhances cell invasion and metastases [18, 32, 33]; we therefore sought to determine whether miR-524-5p also affects cell migration. Cell motility was monitored by a wound-healing assay. Actinomycin D was co-treated with miR-524-5p in cells to prevent the proliferation of cells while we monitored wound closure. We used a non-toxic concentration of actinomycin D that was sufficient to inhibit proliferation (Supplementary Figure 3A and 3B). Wound closure was decreased in miR-524-5p-transfected SK-Mel-19 cells but not in control miR-transfected cells at 48 hours (Figure 6A) or at 24 hours in A375 cells (Figure 6B). Furthermore, we measured the infiltration of melanoma cells using a transwell migration analysis and observed that the miR-524-5p mimic led to a decrease in the migration of SK-Mel-19 and A375 cells relative to control oligonucleotides (Figure 6C and 6D). Similar results were also observed for Malme-3M cells (Supplementary Figure 3C and 3D). Taken together, these results indicate that miR-524-5p exhibits a suppressive function in cancer cell growth, proliferation, and migration.

### miR-524-5p slows the progression of melanoma in mice

**Figure 7:**
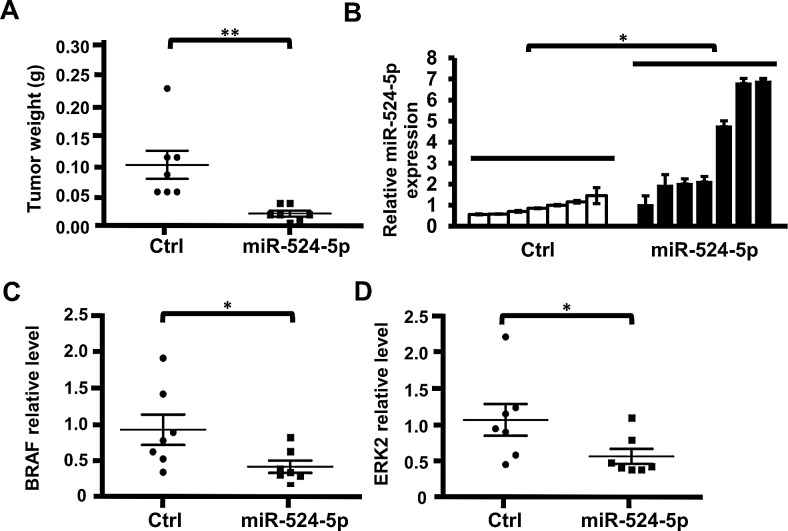
miR-524-5p suppresses the tumorigenicity of melanoma cells *in vivo* (A) SK-Mel-19 cells harboring either the pre-mir-524-5p expression plasmid or control vector (Ctrl) were implanted in each thigh of a nude mouse. After 3 weeks, the tumor weight was measured. (Student's *t*- test: ^**^ *p*< 0.01). (B) Total RNA was extracted from each tumor, and the expression levels of miR-524-5p were detected by qRT-PCR analysis. (C) and (D) The relative quantification results of protein levels of BRAF or ERK2 in each mouse tumor. The level of BRAF or ERK2 was normalized to GAPDH by ImageJ software analysis (Student's *t*- test: ^*^ *p*< 0.05). Western blotting was used to monitor the protein expression levels of BRAF, ERK1/2 and GAPDH in each tumor (Supplementary Figure 4).

To further determine the effect of miR-524-5p on the progression of MAPK/ERK- dependent tumors *in vivo*, SK-Mel-19 cells transiently transfected with the miR-524-5p or negative control plasmid were subcutaneously injected into SCID mice, followed by the observation of xenograft growth. After 3 weeks, the mice were sacrificed, and the tumors were weighed (Figure 7A). The expression of miR-524-5p in each tumor was measured by quantitative real-time PCR (Figure 7B). The results showed that the introduction of miR-524-5p into SK-Mel-19 cells led to a significant reduction in tumor weight. The average tumor weight was reduced by approximately 5-fold in the miR-524-5p-expressing group compared with the control group. In addition, the average protein levels of BRAF or ERK2 in mice in the miR-524-5p-expression group were approximately 2-fold lower than the control group (Figure 6C and 6D, and Supplementary Figure 4). Thus, our experimental data suggest that miR-524-5p has a crucial role in suppressing the growth of melanoma cells *in vivo*.

## DISCUSSION

**Figure 8:**
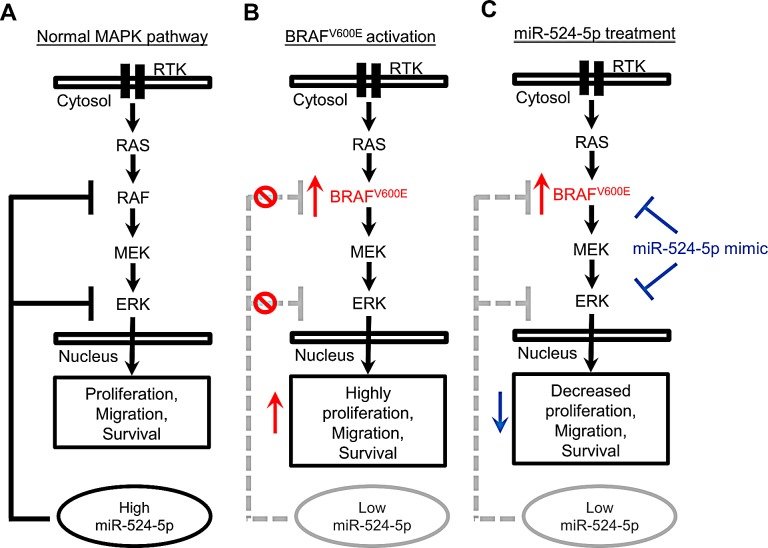
The model of miR-524-5p in MAPK/ERK signaling (A) In wild-type BRAF cells, the activity of MAPK/ERK signaling and the expression of miR-524-5p reached a steady state, resulting in the balance of cell proliferation, migration, and survival. In contrast, (B) MAPK/ERK signaling is highly activated in mutated BRAF cells, but the expression of miR-524-5p is significantly decreased, resulting in the enhancement of cell proliferation, migration, and survival. Moreover, (C) the overexpression of miR-524-5p in mutated BRAF cells significantly suppresses MAPK/ERK activity and reduces cell proliferation, migration, and survival.

There are several ways to regulate MAPK/ERK signaling. The presence of negative-phosphorylation sites on the individual kinases in the MAPK/ERK signaling pathway allows both the fine-tuning and termination of the signal via regulatory feedback loops acting at multiple levels [34-36]. Furthermore, this signaling pathway may induce the expression of proteins that negatively regulate the pathway [37]. In addition to known posttranslational modifications and regulation by proteins that modulate MAPK/ERK signaling, miRNAs represent another group of regulators. Our findings demonstrated that the expression level of miR-524-5p was decreased when the MAPK/ERK pathway was highly activated; conversely, inhibiting MAPK/ERK signaling led to increased miR-524-5p expression. These observations suggest that miR-524-5p is a downstream target of MAPK/ERK signaling. Intriguingly, miR-524-5p also possesses the ability to inhibit MAPK/ERK signaling through the down-regulation of pathway components BRAF and ERK2. Taken together, these results suggest a feedback-loop regulatory mechanism involving miR-524-5p and MAPK/ERK signaling. In our current proposed model, when the miR-524-5p expression level and the activity of MAPK/ERK signaling reach a balance, the cells maintain a steady state (Figure 8A). Continuously highly activated MAPK/ERK signaling lowers the expression of miR-524-5p, rendering it unable to inhibit MAPK/ERK activity and causing the cells to exhibit high proliferation and migration properties (Figure 8B). In contrast, miR-524-5p overexpression inhibits the expression level of BRAF and ERK2 and suppresses MAPK/ERK activity, leading to reduced cell proliferation and migration events (Figure 8C).

A number of miRNAs have been reported to be associated with the MAPK/ERK pathway in different experimental systems and tumors [38-43]. Several of these miRNAs also appeared in our screen with similar deregulation. In cells with an activated MAPK/ERK pathway, the expression levels of let-7a, miR-10, miR-22, miR-26, miR-34, and miR-125a were lower, and those of miR-20, miR-25, and miR-135b, were higher (Supplementary Table 1). Some of these miRNAs have been reported to directly target components of MAPK/ERK signaling, but most appear to target the downstream players of MAPK/ERK signaling, such as proteins regulating the cell cycle and migration. Our study is the first to report that a miRNA can target BRAF; miR-524-5p possesses a dual role in targeting both BRAF and ERK2 in the MAPK/ERK pathway. To our knowledge, this is first report to show that a single miRNA harbors this property. These findings indicate that a concerted miRNA regulatory network is capable of both responding to and inhibiting mitogenic signals.

In our miRNA profiling array, 97 miRNAs were suppressed, and 5 miRNAs were overexpressed in cells with highly activated for MAPK/ERK signaling (Supplementary Table 1), indicating that activating the MAPK/ERK pathway predominantly suppresses miRNA expression. It has been documented that MAPK/ERK could influence the expression of miRNAs via transcription factors. For example, the MAPK/ERK signaling-mediated transcriptional factor Myc has been implicated in melanoma progression and was recently found to function in the widespread suppression of miRNAs [44]. It is possible that the suppression of miRNA expression in melanoma cells with active MAPK/ERK signaling results from Myc-mediated repression or other MAPK-dependent transcription factors. In addition to affecting the activity of transcription factors, MAPK/ERK signaling can also influence components of the miRNA generating complex, e.g., TRBP via its phosphorylation [42].

BRAF inhibitors such as vemurafenib have been associated with some degree of tumor regression in a large percentage of BRAF mutant patients [45]. However, the majority of these patients acquire resistance to BRAF inhibitors, and the mechanisms of this resistance have been elucidated [36]. Reports have shown that the inhibition of MAPK/ERK signaling with BRAF inhibitors relieves the inhibitory autophosphorylation of wild-type BRAF and significantly induces RAS activation. This is due to the relief of ERK-dependent feedback and thus the restoration of ERK activity [34, 36]. These events are thought to cause drug resistance and to induce the formation of other tumors, and there has been a drive to design novel therapies that could circumvent this drug resistance. Because a single miRNA can suppress multiple mRNAs, it may not only suppress an oncoprotein but also may overcome the relief of feedback resulting from a specific inhibitor. Therefore, as master regulators, miRNAs might be a better therapeutic agent than specific small-molecule inhibitors. Indeed, it is notable that several therapeutic miRNAs for cancer have entered into clinical trials [46]. Our results indicate that miR-524-5p has potential as a therapeutic miRNA for MAPK/ERK-mutated tumors. However, it needs to be further determined whether miR-524-5p overexpression can prevent the resistance to BRAF inhibitors. Recently, it has been described that miR-524-5p can modulate the Notch signaling pathway by directly targeting Jagged-1 and Hes-1 in glioma [47]. Interestingly, the MAPK/ERK signaling pathway regulates the activation of Notch signaling through TRB3 in breast cancer [48]. These findings suggest that the overexpression of miR-524-5p may be therapeutically useful for basal-like subtype breast cancer because it can reduce the activity of both the MAPK and Notch signaling pathways.

In summary, we investigated the role of miR-524-5p involved in melanoma. Our study provided new insight into the mechanism by which miR-524-5p inhibits the MAPK/ERK pathway through the down-regulation of BRAF and ERK2, leading to prohibition of tumor growth. Our finding suggests miR-524-5p is a tumor suppressor miRNA and may serve as a potent therapeutic candidate in melanoma treatment.

## MATERIALS AND METHODS

### Cell Culture and Reagents

The HEK293, Malme-3M, Malme-3 and A375 cell lines were obtained from American Type Culture Collection, and SK-Mel-19 was kindly provided by Dr. Neal Rosen (Memorial Sloan-Kettering Cancer Center). The cancer cell lines derived from melanoma (SK-Mel-19, A375 and Malme-3M) were maintained in RPMI-1640 medium (Gibco) supplemented with 10% heat-inactivated fetal bovine serum (Biological Industries), 50 U/ml penicillin, and 50 μg/ml streptomycin at 37°C with 5% CO_2_. The Malme-3 cells, which were derived from skin fibroblasts, were grown in McCoy's 5a Medium (Gibco) supplemented with 20% heat-inactivated FBS, 50 U/ml penicillin, and 50 μg/ml streptomycin at 37°C with 5% CO_2_. The human embryonic kidney cell line HEK293 was maintained in Dulbecco's modified Eagle's medium (Gibco) supplemented with 5% heat-inactivated fetal bovine serum, 50 U/ml penicillin, and 50 μg/ml streptomycin at 37°C with 5% CO_2_. The MEK inhibitors, PD325901, PLX4032 (Selleck) and U0126 (Sigma-Aldrich), and actinomycin D (Enzo) were dissolved in DMSO as stock solutions and stored at −20°C.

### Oligonucleotides and plasmids

The miR-524-5p mimic (miR-524-5p), negative control (NC), and miR-524-5p inhibitor (anti-miR-524-5p) were purchased from Ambion Inc. The 3′UTR was cloned downstream of the luciferase gene to generate pReporter-WT. The plasmids of reporters for WT BRAF and reporter-ERK2 post-transcriptional regulation were constructed as follows. A 580-bp fragment of the BRAF 3′UTR (pMirTarget, Origene) and a 3950-bp fragment of the ERK2 3′UTR (MGC104558, Mammalian Gene Collection, NIH) were amplified then subcloned into the pMIR control vector (Ambion) at the 5′ *Spe*I and 3′ *Hind*III sites using the In-fusion PCR Cloning System (Clontech). A mutant construct of the 3′UTR, named pReporter-MT BRAF, carrying a deletion of six nucleotides within the core seed sequence of possible miR-524-5p binding sites, was generated using site-direct QuikChange XII mutagenesis (Stratagene). pGLO Reporter BRAF WT and pGLO Reporter BRAF MT were generated from pReporter-WT BRAF and pReporter-MT BRAF individually. 3′UTR fragments of BRAF were amplified then subcloned into the pmirGLO vector (Promega) at the 5′ *Nhe*I and 3′ *Xba*I sites by the In-fusion PCR Cloning System (Clontech). A mutant construct of the ERK2 3′UTR, named pReporter ERK2 MT, contained the 2702 bp at the 5′ end fragment of ERK 3′ UTR, which carried a deletion of potential 6^th^ to 8^th^ target sites of miR-524-5p within ERK2 3′UTR sequence. The expression plasmid of the ERK2 coding sequence was constructed from a 1083-bp fragment of the ERK2 cDNA (MGC104558, Mammalian Gene Collection, NIH). The fragment was amplified and then subcloned into the pcDNA3.1 (+) vector (Invitrogene) at the 5′ *Bam*HI and 3′ *Eco*RI sites using the In-fusion PCR Cloning System. All primers are listed in Supplementary Table 2.

### Cell transfection

RNA oligonucleotides were transfected using Lipofectamine RNAiMAX (Invitrogen, CA, USA). Final concentrations of 10 nM miR-524-5p for SK-Mel-19, 30 nM miR-524-5p for A375 and Malme-3M, and 10 nM anti-miR-524-5p for HEK293 were used, unless otherwise indicated. The RNA transfection efficiency was approximately 70–80%, and the presence of miRNA persisted for at least 2 days. X-tremeGENE HP (Roche, IN, USA) was used for the transfection of plasmid DNA. When we co-transfected plasmid DNA and RNA oligonucleotides, the plasmid transfection was processed first for 4 hours, and then we performed the RNA oligonucleotides transfection.

### TaqMan miRNA arrays

TaqMan microRNA arrays (384-Well Fluidic Cards) (Applied Biosystems) were used to profile 381 mature miRNAs. Each card contains a mammalian U6 snRNA assay repeated 4 times, and an assay of non-mammalian *ath-miR-159a* was performed as a process control. RNA (300 ng) from each sample was converted into cDNA using Megaplex RT Primers, TaqMan miRNA RT Kit, and Megaplex PreAmp primers (Applied Biosystems). cDNAs were mixed with TaqMan Universal PCR Master Mix and then loaded into the TaqMan miRNA fluidic cards according to the manufacturer's instructions. The cards were processed using an Applied Biosystems ViiA 7 real-time PCR instrument equipped with a heating block for the fluidic card using universal cycling conditions.

### qRT–PCR

Total RNA was extracted from the cells (or tumor tissues) using mirVana™ miRNA Isolation Kit (Ambion) according to the manufacturer's protocol. miR-524-5p and RNU48 cDNA were synthesized by TaqMan PreAmp Master Mix Kit and TaqMan MicroRNA Assays (Applied Biosystems) according to the manufacturer's instructions to quantify microRNA expression. Relative expression of target microRNAs was calculated using the ^ΔΔ^Ct method and normalized to RNU48.

### Western blot analysis

Cells were washed in phosphate-buffered saline (PBS), and proteins were extracted in NP-40 buffer (50 mM Tris-HCl pH 7.5, 150 mM NaCl, 1% NP-40, 10% glycerol, 1 mM EDTA). The cell lysates were then resolved by electrophoresis through SDS-polyacrylamide gels, and the proteins were electrotransferred onto PVDF membranes. Milk or BSA-blocked blots were incubated with primary antibodies at 4°C overnight and then incubated with horseradish peroxidase-conjugated (HRP) secondary antibodies (Cell Signaling). The proteins were detected using the enhanced chemiluminescence system: Western Blotting Detection Reagents (LumonataTM Classico/Forte Western HPR substrate). The primary antibodies against p42/44 MAPK, phospho-p42/44 MAPK, phospho-MEK, phospho-RSK, cleaved PARP, and GAPDH were obtained from Cell Signaling. Antibodies against BRAF, caspase-3, cyclin D1, β-galactosidase and luciferase were obtained from Santa Cruz. The anti-MEK1/2 antibody was obtained from GeneTex.

### microRNA target prediction

microRNA.org (http://www.microrna.org/microrna/home.do) was used to predict miRNA targets.

### Luciferase reporter gene assay

A total of 3×10^5^ HEK293 cells were seeded in 6-well plates. In the BRAF 3′UTR luciferase reporter assay, 1 μg of β-galactosidase plasmid, used as the transfection efficiency control, was co-transfected with either 1 μg of pReporter, pReporter-WT-BRAF, or pReporter-MT-BRAF. After 4 hours, the cells were transfected with 10 nM miR-524-5p or the negative control. Another BRAF 3′UTR luciferase reporter, 2 μg of pGLO Reporter-WT BRAF 3′UTR or pGLO Reporter-MT BRAF 3′UTR was transected into HEK293 cells. In the ERK2 3′UTR luciferase reporter assay, 1 μg of β-galactosidase plasmid was co-transfected with either 1 μg of pReporter or pReporter-WT ERK2. After 4 hours, the cells were transfected with 10 nM miR-524-5p and 20 nM anti-miR-524-5p or the negative control. The mimic microRNAs and plasmids were transfected using Lipofectamine RNAiMAX (Invitrogen) and X-tremegene HP (Roche), respectively. The cells were lysed and assayed for protein expression after 48 hours of transfection.

### Analysis of cell proliferation

A total of 3×10^5^ cells were plated in 6-well plates and transfected with 10 nM mimic miR-524-5p or the negative control. After 48 hours, cell proliferation was determined by AlamarBlue assays (Invitrogen) according to the manufacturer's instructions. The fluorescence values were measured at excitation wavelengths of 530-560 nm and an emission wavelength of 590 nm. All measured values were detected by Synergy HT (BioTek, VT, USA).

### Wound healing assay

A total of 2×10^5^ cells were seeded in 12-well plates and transfected with mimic miR-524-5p or negative control. At 48 hours post-transfection, the cell layer was scratched with a 10-μl pipette tip and followed by treatment with 0.01 μg/ml actinomycin D to inhibit the cell proliferation. The ability of the cells to close the wound was photographed.

### Migration assay

A 24-well transwell plate (8-μm pore size, Corning) was used to measure the migratory ability of the cells. A total of 2×10^5^ cells were plated in the top chamber lined with a non-coated membrane in penicillin and streptomycin-free medium. After 2 hours, the cells were transfected with 10 nM mimic hsa-miR-524-5p or the negative control. After incubation for 48 hours, the migratory cells on the underside of the membrane were stained with 0.1% crystal violet for 5 minutes and washed with H_2_O; the membrane was scanned using an EPSON V750 PRO scanner. The crystal violet was destained with methanol for 15 minutes, and the absorbance values were measured at OD_570_. All measured values were detected by Synergy HT (BioTek).

### Analysis of colony formation

For clonogenicity analysis, 3×10^5^ cells were seeded in 6-well plates and transfected with 2 μg miR-524 plasmid or 2 μg pCHD cDNA plasmid as a control (System Biosciences). At 48 hours post-transfection, 8×10^3^ viable transfected cells were placed in 12-well plates with a suitable percentage of soft agar (0.6% bottom layer, 0.3% top layer) and maintained in complete medium for three to four weeks. The colonies were stained with 0.005% crystal violet for 5 minutes and photographed.

### Xenograft

All of the experimental procedures involving animals were performed according to the institutional ethical guidelines for animal experimentation and were approved by the Animal Ethics Committee of Academia Sinica and National Central University (Taiwan). A total of 2×10^6^ cells were plated in 10-cm dishes and transfected with 5 μg miR-524 plasmid or 5 μg pCHD cDNA plasmid as a control using X-tremegene HP (Roche). After 48 hours of incubation, 2.5×10^6^ viable cells were injected subcutaneously into 6-week-old male NOD/SCID mice, 7 per group. Tumor growth was measured at 6 days after injection and then every 7 days. At 4 weeks after injection, the mice were sacrificed, and the tumors were weighed after necropsy.

### Statistical analysis

Each experiment was repeated at least three times. Numerical data are presented as the mean ± s.d. Unless otherwise indicated, the differences between two groups were analyzed using Student's t-test (two-tailed). The differences were considered statistically significant at P<0.05. All of the statistical analyses were performed using SigmaPlot software 10.0 (SYSTAT).

## SUPPLEMENTARY MATERIALS AND METHODS, FIGURES AND TABLES


