# Supplementary material for: miR-524-5p suppresses the growth of oncogenic BRAF melanoma by targeting BRAF and ERK2
Source: Oncotarget. 2014 Sep 8;5(19):9444–59. doi: 10.18632/oncotarget.2452 (PMC4253445; doi:10.18632/oncotarget.2452)
Supplement: Supplementary file 1 [file oncotarget-05-9444-s001.pdf]

miR-524-5p suppresses the growth of oncogenic BRAF melanoma by targeting BRAF and ERK2

Supplementary Materials and Methods

Cells and Reagents

SK-Mel-187 was kindly provided by Dr. Neal Rosen (Memorial Sloan-Kettering Cancer Center, NY, USA) and cells were maintained in RPMI-1640 medium (Gibco, CA, USA) supplemented with heat inactivated 10% fetal bovine serum (Biological Industries), 100 U/ml penicillin and 100 mg/ml streptomycin at 37°C with 5% CO_2. Actinomycin D (Enzo, Taipei, Taiwan) were dissolved in DMSO as stock solutions and stored at -20°C.

Supplementary Table 1-1, 1-2 and 1-3

The expression of miRNAs was determined after quantitative real-time miRNA PCR array to investigate the difference between of expression in Malme-3 and Malme-3M. Total 216 miRNAs expression profile was listed in this Table. The fold change in the expression (the value in Malme-3/the value in Malme-3M) was calculated for each miRNA.

Supplementary Table 1-1

microRNA name	Fold change	microRNA name	Fold change	microRNA name	Fold change
hsa-miR-31	487485.130	hsa-miR-199b	735.203	hsa-miR-615-5p	48.235
hsa-miR-337-5p	299458.730	hsa-miR-518f	653.481	hsa-miR-520b	46.916
hsa-miR-376c	184850.385	hsa-miR-524-5p	616.946	hsa-let-7c	46.078
hsa-miR-376a	147668.145	hsa-miR-486-3p	566.527	hsa-miR-152	45.412
hsa-miR-214	110447.436	hsa-miR-485-3p	563.785	hsa-miR-100	45.223
hsa-miR-382	96885.786	hsa-miR-618	494.559	hsa-miR-99a	43.411
hsa-miR-127	55492.242	hsa-miR-708	320.016	hsa-miR-142-3p	40.392
hsa-miR-302c	54766.318	hsa-miR-202	283.263	hsa-miR-27a	40.364
hsa-miR-886-5p	41218.415	hsa-miR-196b	277.626	hsa-miR-149	35.977
hsa-miR-411	38752.421	hsa-miR-221	252.126	hsa-miR-423-5p	34.824
hsa-miR-886-3p	38192.425	hsa-miR-125a-3p	178.775	hsa-miR-23b	31.956
hsa-miR-410	23445.094	hsa-miR-511	128.712	hsa-miR-34c	31.669
hsa-miR-487b	16147.243	hsa-miR-548c	128.444	hsa-miR-27b	29.263
hsa-miR-493	15980.212	hsa-miR-22	99.526	hsa-miR-193a-5p	28.681
hsa-miR-198	11252.831	hsa-miR-490	81.515	hsa-miR-361	27.455
hsa-miR-539	8439.848	hsa-miR-136	81.009	hsa-miR-887	27.115
hsa-miR-433	7491.300	hsa-miR-125b	79.617	hsa-miR-339-3p	25.176
hsa-miR-494	6458.573	hsa-miR-210	74.491	hsa-miR-320	23.539
hsa-miR-483-5p	2646.737	hsa-miR-503	71.556	hsa-let-7a	22.285
hsa-miR-145	1782.887	hsa-miR-342-5p	69.023	hsa-miR-744	22.116
hsa-miR-199a-3p	1634.914	hsa-miR-517a	68.072	hsa-let-7e	21.706
hsa-miR-323-3p	1482.689	hsa-miR-542-5p	67.837	hsa-miR-218	20.322
hsa-miR-655	1363.405	hsa-miR-519d	62.336	hsa-miR-518d	19.959
hsa-miR-375	1292.547	hsa-miR-193a-3p	54.682	hsa-miR-155	19.508
hsa-miR-143	780.362	hsa-let-7b	50.563	hsa-miR-220b	18.278

Supplementary Table 1-2

microRNA name	Fold change	microRNA name	Fold change	microRNA name	Fold change
hsa-miR-23a	17.594	hsa-miR-891a	9.299	hsa-miR-628-5p	5.400
hsa-miR-24	16.877	hsa-miR-190	9.222	hsa-miR-181a	4.780
hsa-miR-505	16.427	hsa-miR-98	9.082	hsa-miR-28-3p	4.754
hsa-miR-150	16.257	hsa-miR-222	9.082	hsa-miR-26b	4.682
hsa-miR-570	16.156	hsa-miR-186	8.821	hsa-miR-200b	4.426
hsa-miR-627	15.956	hsa-miR-629	8.760	hsa-miR-103	4.414
hsa-miR-487a	15.466	hsa-miR-223	8.545	hsa-miR-130b	4.347
hsa-miR-212	14.908	hsa-miR-424	8.369	hsa-miR-146b	4.299
hsa-let-7d	14.764	hsa-miR-296-3p	8.202	hsa-miR-28	4.031
hsa-miR-215	13.900	hsa-miR-455	8.162	hsa-miR-455-3p	3.986
hsa-miR-636	12.321	hsa-miR-545	7.841	hsa-miR-324-5p	3.891
hsa-miR-518b	11.926	hsa-miR-29c	7.511	hsa-miR-125a-5p	3.848
hsa-miR-576-3p	11.893	hsa-miR-324-3p	7.392	hsa-miR-21	3.635
hsa-miR-193b	11.181	hsa-miR-10a	7.250	hsa-miR-99b	3.618
hsa-let-7f	11.081	hsa-miR-34a	7.220	hsa-miR-501	3.519
hsa-miR-130a	10.830	hsa-miR-141	6.926	hsa-miR-107	3.387
hsa-miR-574-3p	10.659	hsa-miR-185	6.788	hsa-miR-425-5p	3.147
hsa-miR-192	10.527	hsa-miR-181c	6.413	hsa-miR-454	2.821
hsa-let-7g	10.490	hsa-miR-671-3p	6.316	hsa-miR-301b	2.782
hsa-miR-449	10.382	hsa-miR-489	5.913	hsa-miR-362	2.732
hsa-miR-597	10.218	hsa-miR-140-3p	5.873	hsa-miR-10b	2.721
hsa-miR-29a	10.091	hsa-miR-450a	5.768	hsa-miR-29b	2.668
hsa-miR-191	9.931	hsa-miR-579	5.626	hsa-miR-132	2.570
hsa-miR-195	9.487	hsa-miR-148b	5.540	hsa-miR-26a	2.507
hsa-miR-15a	9.441	hsa-miR-486	5.460	hsa-miR-194	2.500

Supplementary Table 1-3

microRNA name	Fold change	microRNA name	Fold change	microRNA name	Fold change
hsa-miR-625	2.481	hsa-miR-95	1.519	hsa-miR-642	0.689
hsa-miR-197	2.478	hsa-miR-374	1.488	hsa-miR-376b	0.674
hsa-miR-542-3p	2.471	hsa-miR-18b	1.468	hsa-miR-342-3p	0.641
hsa-miR-331	2.425	hsa-miR-106b	1.405	hsa-miR-532-3p	0.616
hsa-miR-484	2.326	hsa-miR-330	1.360	hsa-miR-184	0.587
hsa-miR-138	2.204	hsa-miR-101	1.353	hsa-miR-522	0.514
hsa-miR-16	2.046	hsa-miR-500	1.341	hsa-miR-340	0.475
hsa-miR-331-5p	2.045	hsa-miR-17	1.328	hsa-miR-362-3p	0.469
hsa-miR-128a	2.020	hsa-miR-589	1.259	hsa-miR-20b	0.430
hsa-miR-19b	1.957	hsa-miR-139-5p	1.168	hsa-miR-204	0.427
hsa-miR-660	1.913	hsa-miR-19a	1.146	hsa-miR-15b	0.365
hsa-miR-328	1.840	hsa-miR-200c	1.146	hsa-miR-25	0.300
hsa-miR-616	1.803	hsa-miR-502	1.058	hsa-miR-652	0.261
hsa-miR-345	1.728	hsa-miR-92a	1.054	hsa-miR-135b	0.157
hsa-miR-449b	1.676	hsa-miR-18a	1.003	hsa-miR-9	0.150
hsa-miR-30c	1.666	hsa-miR-139-3p	0.892	hsa-miR-299-5p	0.147
hsa-miR-32	1.620	hsa-miR-296	0.837	hsa-miR-148a	0.107
hsa-miR-301	1.619	hsa-miR-502-3p	0.820	hsa-miR-126	0.094
hsa-miR-590-5p	1.608	hsa-miR-30b	0.742	hsa-miR-135a	0.022
hsa-miR-532	1.593	hsa-miR-422a	0.742	hsa-miR-517b	0.009
hsa-miR-339-5p	1.579	hsa-miR-20a	0.735	hsa-miR-146a	0.007
hsa-miR-106a	1.521	hsa-miR-365	0.706	hsa-miR-211	0.001

Supplementary Table 2: Primers listed above were utilized for constructing plasmids of pReporter-WT BRAF, pReporter--MT BRAF, pGLO Reporter--BRAF WT, pGLO Reporter--BRAF MT, pReporter--ERK2 WT, pReporter--ERK2 MT and ERK2 expression plasmid Primers.

Primer name	Sequence (5'→3')
BRAF 3'UTR F primer (pMIR luciferase reporter)	AAAGCTGCGCACTAGTTATGGTGCGTTTCCTGTCCACTGAAAC
BRAF 3'UTR R primer (pMIR luciferase reporter)	ATCCTTTATTAAGCTTCAAGGAAATAAAAGACATCCACATTTTCCAAATTG
BRAF 3'UTR F primer (GLO luciferase reporter)	AAACGAGCTCGCTAGCTATGGTGCGTTTCCTGTCCACTGAAAC
BRAF 3'UTR R primer (GLO luciferase reporter)	GCAGGTCGACTCTAGACAAGGAAATAAAAGACATCCACATTTTCCAAATTG
ERK2 3'UTR F primer	AAAGCTGCGCACTAGTATTTGTCAGGACAAGGGCTCAGAGGACT
ERK2 3'UTR R primer	ATCCTTTATTAAGCTTGTTATTTTGGTAATACAGGGACACAATTTAATGATTCTA
BRAF 3'UTR del F primer (pMIR luciferase reporter)	AGTTTACTACTTAAAATAACCAAGTGAATGACCCGGAACAGAAAGTA
BRAF 3'UTR del R primer (pMIR luciferase reporter)	TACTTTCTGTTCCGGGTCATTCACTTGGTTATTTTAAGTAGTAAACT
ERK2 3'UTR del F primer	ATCCTTTATTAAGCTTATTAGTCTGAAAGCTTATTG
ERK2 3'UTR del R primer	AAAGCTGCGCACTAGATTTGTCAGGACAAGGGCT
ERK2 CDS F primer	TACCGAGCTCGGATCCATGGCGGCGGCGGCGGCG
ERK2 CDS R primer	GATATCTGCAGAATTCTTAAGATCTGTATCCTGGCT

Supplementary figure 1

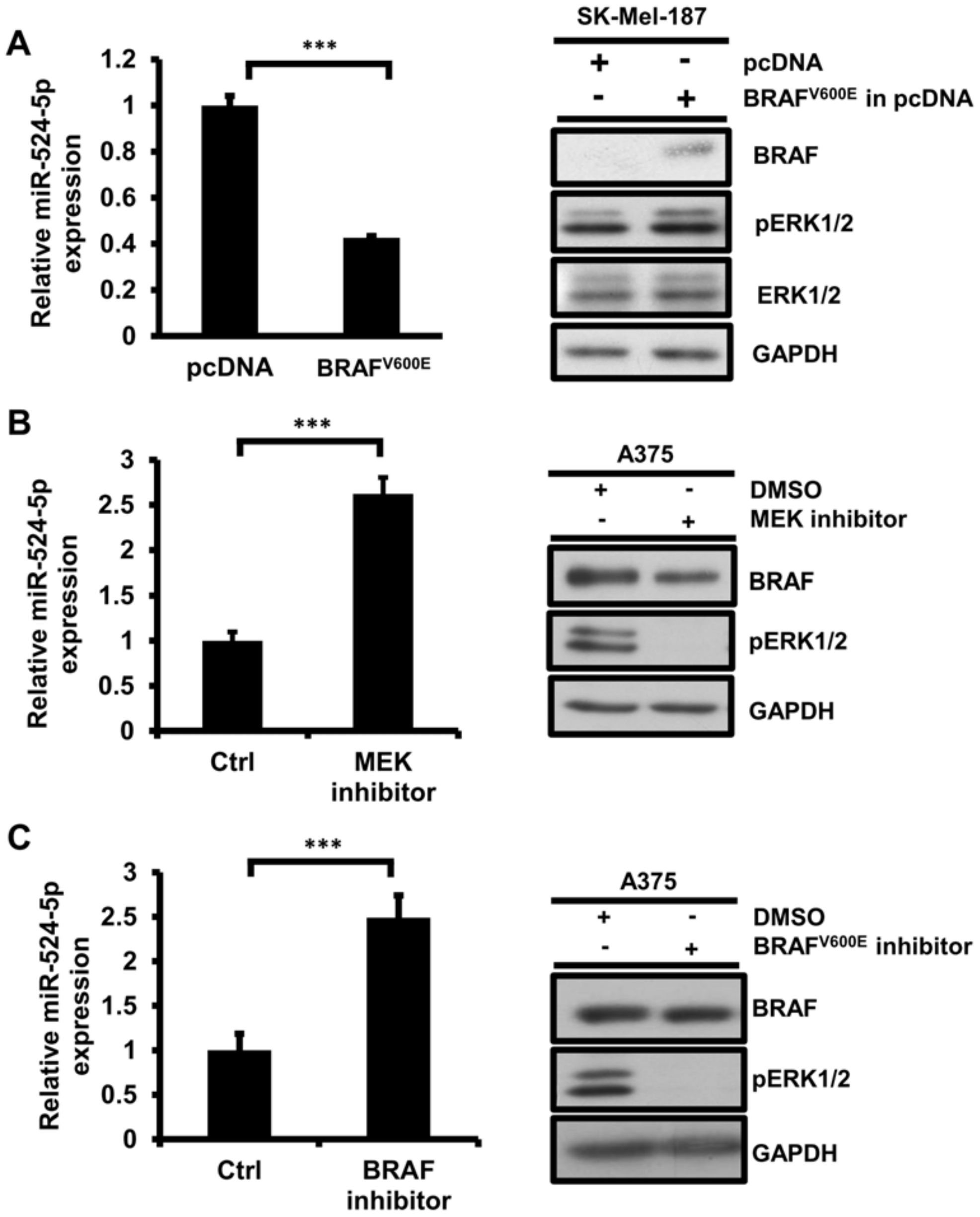

Supplementary Figure 1 : Left panel, the expression level of miR-524-5p is detected by quantitative RT-PCR (normalized to RNU48). Each value represents the average from triplicates. Error bars mark the standard deviations. (Student's *t*- test: *** $p< 0.001$) Right panel, the activity of MAPK/ERK pathway was measured by Western analysis to detect protein levels of BRAF and phospho-ERK, and ERK. (A) Over-expressing mutated V600E BRAF in SK-Mel-187 cells for 48 hours results in activated MAPK pathway. (B) A375 cells were treated with or without 50 nM of MEK1/2 inhibitors PD0325901 for 24 hours, and the cells were collected for analyses. (C) A375 cells were treated with or without 5 μM of V600E BRAF inhibitors PLX4032 for 8 hours, and the cells were collected for analyses.

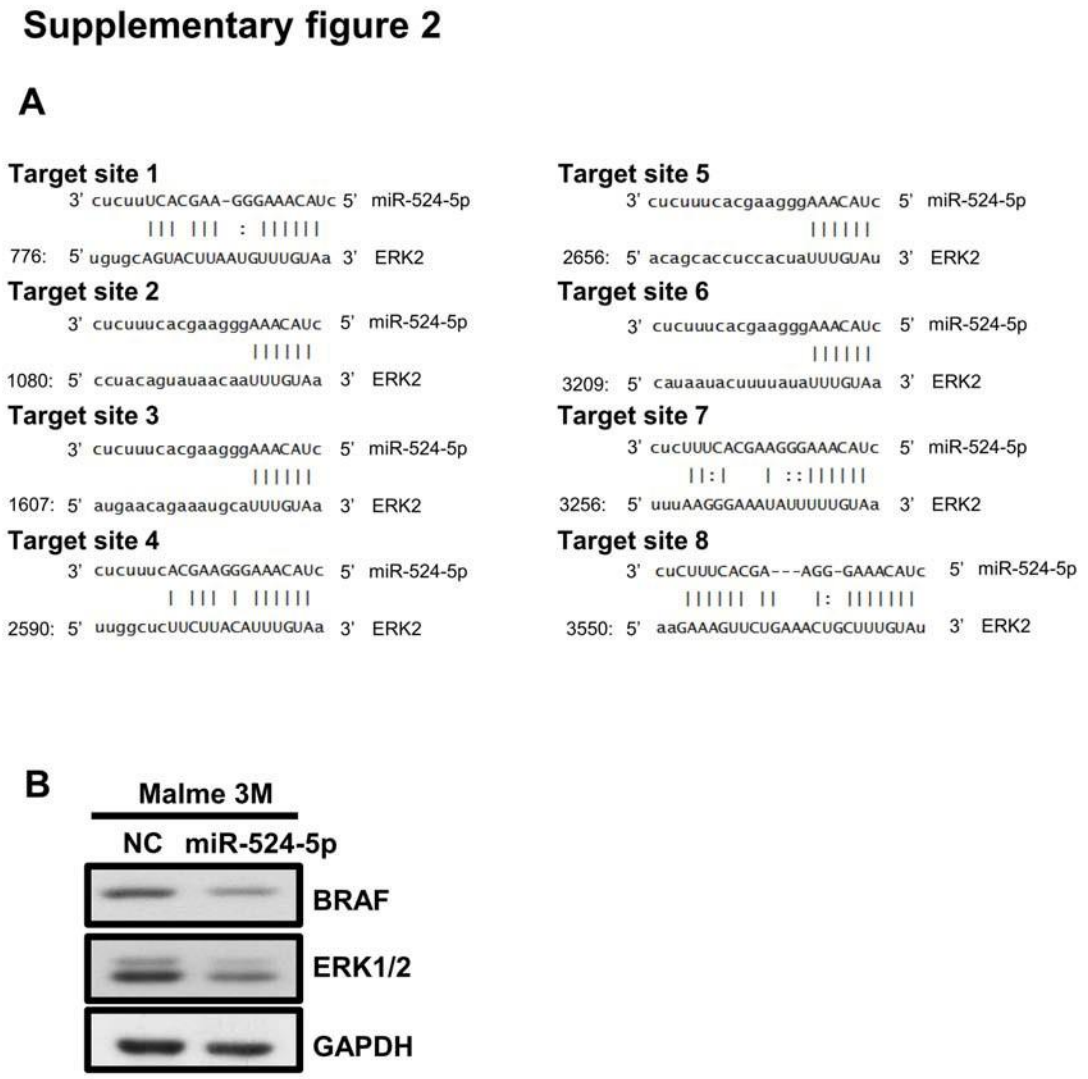

Supplementary Figure 2: (A) Schematic of the ERK2 3'-UTR showing binding sites for miR-524-5p. Eight possible binding sites of miR-524-5p were located in the 3' UTR of ERK2 according to microRNA target prediction. (B) miR-524-5p overexpression suppressed the protein expression of BRAF and ERK2 in Malme-3M cell lines. GAPDH served as an internal control.

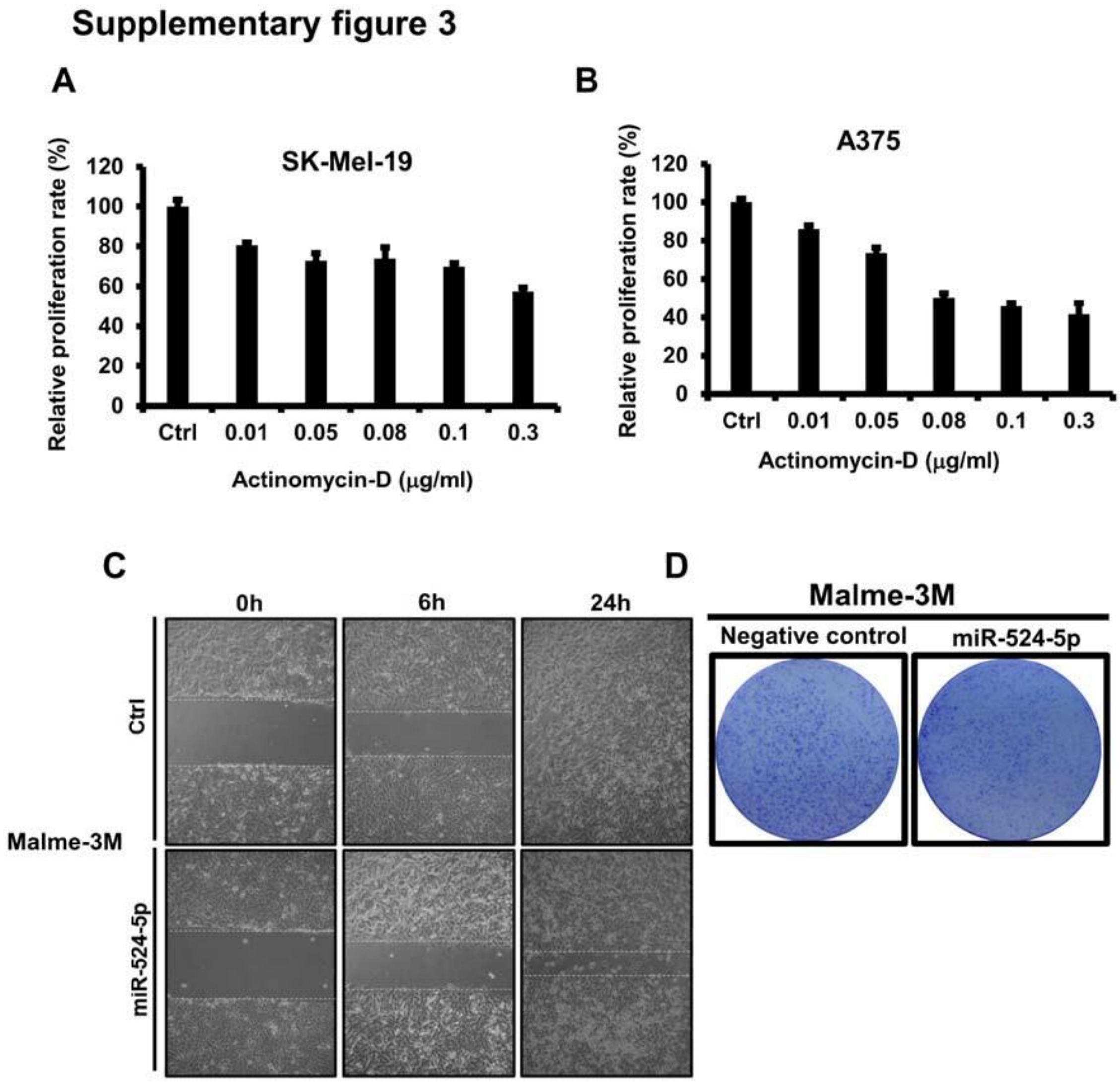

Supplementary Figure 3: (A) and (B) The proliferation activities were detected by the AlamarBlue assay. SK-MEL-19 (A) or A375 (B) cells were treated with different concentrations of actinomycine D for 24 hours and detected their survival activity. (C) and (D) Malme-3M cells were transfected with 30 nM negative control or mimic miR-524-5p. (C) At different time points, the activity of recovery wound healing was observed. Representative micrographs show cells immediately after the scratch 0, 6 and 24 hours. (D) Cells were plated onto transwell filters, and the migration activity was detected after 24 hours by transwell assays.

Supplementary figure 4

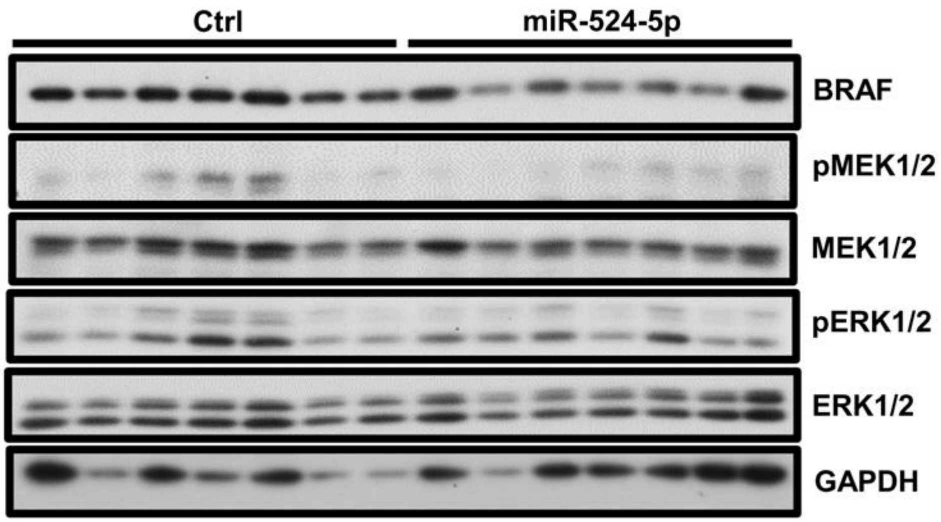

Supplementary Figure 4: Western blotting was used to monitor the protein expression levels of BRAF, pMEK1/2,MEK1/2, pERK, ERK and GAPDH in each tumor.
